# Network centrality and seasonality interact to predict lice load in a social primate

**DOI:** 10.1038/srep22095

**Published:** 2016-02-26

**Authors:** Julie Duboscq, Valeria Romano, Cédric Sueur, Andrew J.J. MacIntosh

**Affiliations:** 1 Centre National de la Recherche Scientifique, Département Ecologie, Physiologie et Ethologie, Strasbourg, France; 2 Université de Strasbourg, Institut Pluridisciplinaire Hubert Curien, Strasbourg, France; 3 Unit of Social Ecology, CP231, Université libre de Bruxelles, Campus Plaine, Brussels, Belgium; 4 Kyoto University Primate Research Institute, Inuyama, Japan; 5 Kyoto University Wildlife Research Center, Kyoto, Japan

## Abstract

Lice are socially-transmitted ectoparasites. Transmission depends upon their host’s degree of contact with conspecifics. While grooming facilitates ectoparasite transmission via body contact, it also constrains their spread through parasite removal. We investigated relations between parasite burden and sociality in female Japanese macaques following two opposing predictions: i) central females in contact/grooming networks harbour more lice, related to their numerous contacts; ii) central females harbour fewer lice, related to receiving more grooming. We estimated lice load non-invasively using the conspicuous louse egg-picking behaviour performed by macaques during grooming. We tested for covariation in several centrality measures and lice load, controlling for season, female reproductive state and dominance rank. Results show that the interaction between degree centrality (number of partners) and seasonality predicted lice load: females interacting with more partners had fewer lice than those interacting with fewer partners in winter and summer, whereas there was no relationship between lice load and centrality in spring and fall. This is counter to the prediction that increased contact leads to greater louse burden but fits the prediction that social grooming limits louse burden. Interactions between environmental seasonality and both parasite and host biology appeared to mediate the role of social processes in louse burden.

Many parasites, i.e. organisms that live and feed exclusively within or on other living organisms (hosts), are socially-transmitted, either directly through contact between individuals or indirectly through spatial overlap and resource sharing^1^. Risk of infection in social individuals can therefore depend partly upon the nature of their social interactions, making the risk of disease and pathogen transmission one of the major costs of sociality^2^. Highly social hosts are expected to encounter a more abundant and diverse parasite community than less social hosts, and thus to exert stronger influence on the transmission of parasites through their social networks^1^. Thus the heterogeneity and diversity of contacts between hosts must be considered when tracking parasite/disease transmission and attempting to understand infection risk^3^,^4^.

From this perspective, social network analysis (SNA) provides a useful tool that captures such heterogeneity by taking into account direct and indirect connections (edges) between individuals (nodes), allowing for multilevel analyses from individuals to populations^3^. For example, the number of connections an individual has (network degree) and their combined ‘weight’ (network strength) can be used to assess an individual’s risk of direct exposure to pathogens/parasites from social conspecifics, while other indices such as eigenvector centrality extend estimation of exposure risk to include the neighbourhood of an individual’s neighbours^3^,^5^. The use of SNA in epidemiological studies has also highlighted that social transmission of pathogens can be dynamic because host sociality itself is intrinsically dynamic^3^ and related to various factors which must also be taken into account such as environmental seasonality, age, sex, reproductive state, energetic needs and/or preferential attachment related to dominance rank, kinship, or friendship^2^,^6^.

One common type of network relevant to social animals derives from social grooming (allo-body-care/allo-grooming/allo-preening, hereafter grooming). Grooming is often highlighted as a mechanism of establishing and maintaining group cohesion and social bonds^7^,^8^, and at least in non-human primates, its frequency has been linked to kinship, dominance rank and access to resources^9^,^10^. In many social animals, grooming is also studied with respect to its direct and indirect health benefits: in addition to reducing physiological stress and releasing endorphins (“relaxing” hormones)^11^,^12^,^13^, social grooming retains the original (hygienic) function of self-grooming which evolved to remove dirt and other debris, ectoparasites and/or dead skin. Evidence for this hygienic function is widespread in animal societies^14^,^15^,^16^. It is further supported by the preference of groomers to groom body parts likely infested with ectoparasites because they are not easily accessible to the individuals themselves (e.g. head, shoulders)^16^,^17^,^18^, and by the finding that preventing grooming through physical incapacitation or social isolation leads to sharp increases in ectoparasite loads^19^,^20^.

One common ectoparasite of mammals is the louse, which feeds on blood and requires a host during each stage of its life cycle. Adult lice typically lay one to several eggs (nitts) every few days, which are glued to the base of a hair. Like many organisms, lice respond to environmental conditions (e.g. temperature, humidity). In ungulates, for instance, adult louse populations peak in spring and drop in summer^20^,^21^. Lice are also susceptible to their host’s physical condition (e.g. hormonal changes), which they perceive through blood meals (e.g.)^21^,^22^,^23^. Thus, louse reproduction can be triggered or hindered by that of their hosts^20^. Lice are further affected by changes in the pelage of their hosts–their habitat–such as those induced by moulting (sequential hair replacement) or shearing (e.g. in domestic sheep)^23^. All of these are relevant to the extent to which lice affect their hosts. Direct effects of infestation include activation of skin allergic reactions such as dermatitis or pruritis^20^, which have pronounced effects on an animal’s body condition, e.g. hair/feather quality^20^, and ultimately its fitness^20^. Indirect effects include their potential to act as vectors or intermediate hosts of many pathogens (e.g., *Rickettsia prowazekii*, *Bartonella quintana*, and *B. recurrentis*^24^; *Rickettsia*, *Anaplasma*, and *Bartonella* spp. in various animals^20^,^25^,^26^, including primates^27^).

Lice and their mammalian hosts are thus a good host-parasite system to study the likelihood or risk of disease/parasite transmission within host social networks^28^. Body contact, including grooming, provides an opportunity for these parasites to transfer from one host to the next. The host contact network structure is therefore paramount in patterns of louse transmission and even population viability, since lice are largely host specific, have a direct life cycle, and may not survive more than a few hours away from their host^20^. At the same time, much of the body contact observed in many social animals occurs in the context of social grooming, which may simultaneously constrain the spread of such organisms through parasite removal. An individual’s lice load can thus be amplified or constrained by its social contact network, illustrating one of the trade-offs between costs and benefits of being social. Yet, studies investigating the links between network centrality and louse infestation and/or louse-mediated disease are still too few, and these trade-offs thus generally remain poorly understood.

In this study, we investigated the risk of louse infestation within Japanese macaque social networks. The Japanese macaque (*Macaca fuscata fuscata*) is a social primate species living in multi-female multi-male groups, where individuals form linear dominance hierarchies and differentiated affiliative social relationships^29^. Japanese macaques harbour two species of louse (*Pedicinis obtusus* and *P. eurygaster*). Based on quantification of egg and nymphal/adult stages of lice detected on culled macaques, it was estimated that an average-sized macaque could harbour up to 550 louse eggs^17^. This was approximated to represent 230 nymph/adult lice^17^, with usually good correlation between these life stages (at least in domestic sheep)^21^. Thus, the number of louse eggs seems a good estimation of the host louse population^17^. Furthermore, video data analysis showed that 98.9% of what groomers conspicuously pick out and consume from the hair/skin of groomees consists of louse eggs^30^. Body parts estimated to have many louse eggs and associated with higher frequencies of conspicuous louse egg-picking gestures are groomed longer than other body parts^17^,^31^. This louse egg-picking behaviour can thus provide an ideal estimator of lice load among individuals observed under naturalistic conditions. We therefore used the number of louse egg-picking gestures during grooming, controlling for total number of observation records of grooming received, as a proxy for lice load (see Methods).

Specifically, we first assessed the extent of variation in centrality measures and lice load according to ecological and social contexts because host-parasite interactions can be mediated by such contexts^32^. Japanese macaques live in an environment with four distinct seasons and are strict seasonal breeders, with mating seasons occurring between fall and winter and birth seasons occurring between spring and summer (with strong regional variation)^29^. As such, host energetic demands and physiology as well as social contact and proximity networks also change seasonally and seasonal changes in host reproductive activity can induce variation in host immune defence^32^ and social tendencies^33^,^34^,^35^. Individuals furthermore moult seasonally, hair being longest/densest in winter and shortest/sparsest in summer^36^, which may strongly affect louse reproduction and survival but also louse detection during grooming. Host susceptibility to infection can also be related to an individual’s general physical condition^37^. Higher-ranking females in the dominance hierarchy are generally fitter than lower-ranking ones^38^,^39^,^40^,^41^, and they may thus better resist infection^35^,^42^,^43^. Such individual, social and environmental variables, along with the synergies among them, often correlate with variation in parasitism which in turn may influence transmission dynamics and infection risks^32^. As such we predicted that environmental seasonality, host reproductive status and dominance rank would affect host centrality, lice load and their interactions in a way that females, particularly low-ranking ones, may be less social and more prone to lice infestation during reproductive seasons (i.e. winter and summer), periods where lice population should also be either at its maximum or at its minimum due to local conditions (i.e. physiological and hair status of the host).

Then, we tested the relationship between centrality and lice load, accounting for the potential confounding factors presented above (also see Methods). Because body contact provides a transmission opportunity for lice, and thus an infection risk for hosts, yet grooming may constrain louse density through parasite removal, we made two opposing predictions: compared to less central females, 1/ more central females will be most infested with lice due to their higher diversity of interacting partners or their higher frequency of body contact in the network; or 2/ more central females will be least infested with lice because they have their louse eggs removed through grooming by a higher diversity of partners and/or more frequently. Evidence for a positive relationship between centrality and lice load would indicate that despite increasing exposure risk to potentially deleterious parasites, being central still presents advantages. Conversely, a negative relationship would speak in favour of social grooming as an efficient antiparasite strategy that can be exploited by females through their grooming networks. To test prediction 1, we investigated the relationship between estimated lice load and centrality measures based on undirected weighted networks of body contacts, including grooming. To test prediction 2, we looked at whether lice load was related to in-centrality measures derived from directed weighted networks of grooming received. Testing different centrality measures allowed us to investigate whether it is the actual number (or diversity) of partners and/or the actual amount of social contact a female has that influences lice load. First, grooming skills and thus louse egg-picking efficiency significantly vary across individuals^44^. As such, being groomed for long duration by an individual poorly skilled at removing louse eggs can have less influence on lice load than being groomed for short durations by several individuals with varying degree of efficiency at removing louse eggs. Furthermore, female Japanese macaque society is based on strict hierarchical social rules determining who can interact with whom^45^. These social constraints can not only affect the number of partners as much as the frequency of social interactions of an individual, but also the areas of the body individuals have access to: lower-ranking females indeed tend to avoid eye contact when grooming higher-ranking ones, and thus avoid the head and frontal body parts (in bonobos)^46^. Given that lice are unevenly distributed on the body^17^, this may also constrain louse egg removal and having diverse grooming partners may thus be as advantageous as being groomed for long durations.

## Results

Grooming represented 27% (median, range = 14–39%, N = 20) of all activity scans of female Japanese macaques of Koshima during this study. Body contact without grooming represented only 9% (median, range = 1–20%, N = 20) of all scans with body contact. Females were in contact with other adult females in 48% of all scans with body contact (median, range = 4–88%, N = 20), with males in 6% (median, range = 0–61%, N = 20 females and 9 males) and sub-adults, juveniles and infants in 41% (median, range = 1–96%, N = 20 females and 23–31 non-adults). Total louse egg-picking events averaged 129 events per female over the whole study period (median, range: 36–320, N = 20), which represented less than one event per grooming minute-scan (median = 0.77, range = 0.34–2.23, N = 20).

### Variation in centrality and lice load according to seasonal and individual factors

**Figure 1:**
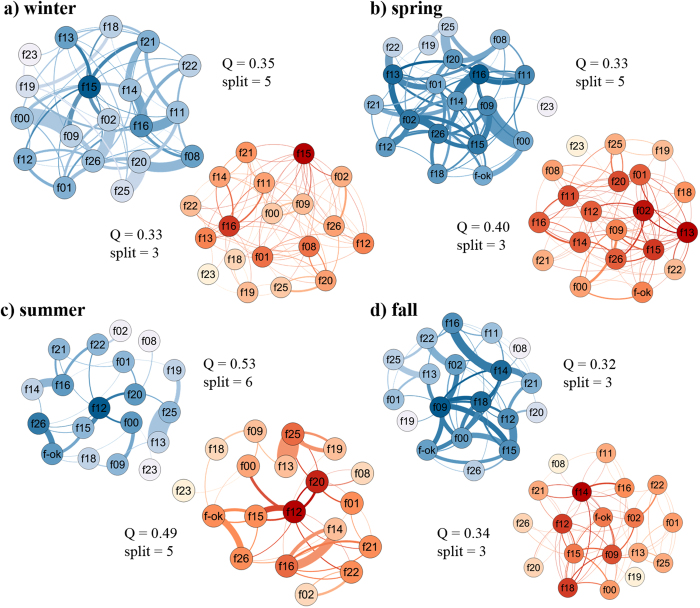
Seasonal variation in contact and grooming networks. Sociograms of weighted contact (in blue) and grooming received (in orange) networks across the four seasons. Node colour shades represent variation in number of connections (degree), the darker the higher, and edge thickness represents the strength of the connection between two nodes, the thicker the stronger. A bidirectional relationship between two nodes is indicated by two edges clockwise. On the side of each network is given the modularity Q as well as the number of communities (or splits) found according to eigenvector centrality (Newman 2006).

**Figure 2:**
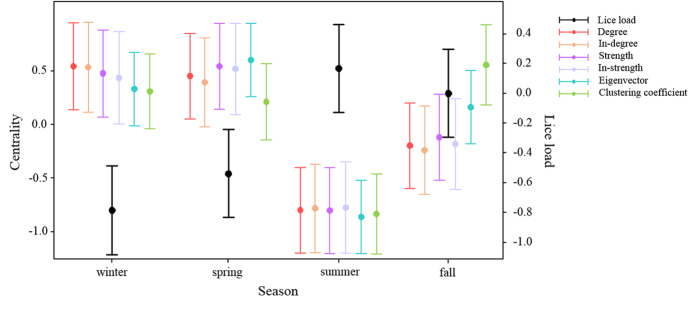
Variation in centrality indices and lice load across seasons. Coefficient plots of GLMMs testing the influence of seasonal and individual factors on centrality measures and lice load (here without the influence of centrality). Circle: coefficient value, bold line: one standard error. Levels of categorical predictors between parentheses indicate those not included in the intercept (the reference level is winter for season, not reproductively active for reproductive state).

The modularity Q of social networks, representing the extent to which a network is partitioned in smaller units, shifted between seasons especially before and after summer where it was highest (Fig. 1). Randomisation tests showed that only the centrality measure degree in the contact network was significantly affected by seasonal and/or individual factors when compared to null models that randomised the network data. Degree in the contact network showed significant variation across seasons (Supplementary Table S2; Figs 1 and 2): it was significantly lower in summer and fall compared to winter and spring (Supplementary Table S2; Fig. 2), meaning that females had significantly more female social partners during the latter than the former seasons.

Lice load also varied across seasons, being higher in summer and fall compared to spring and winter (Supplementary Table S2; Fig. 2). Lice load changed marginally according to the females’ reproductive state, being slightly higher in reproductively active females than others (Supplementary Table S2).

### Testing prediction 1: increased parasitism with increased centrality in contact networks

**Figure 3:**
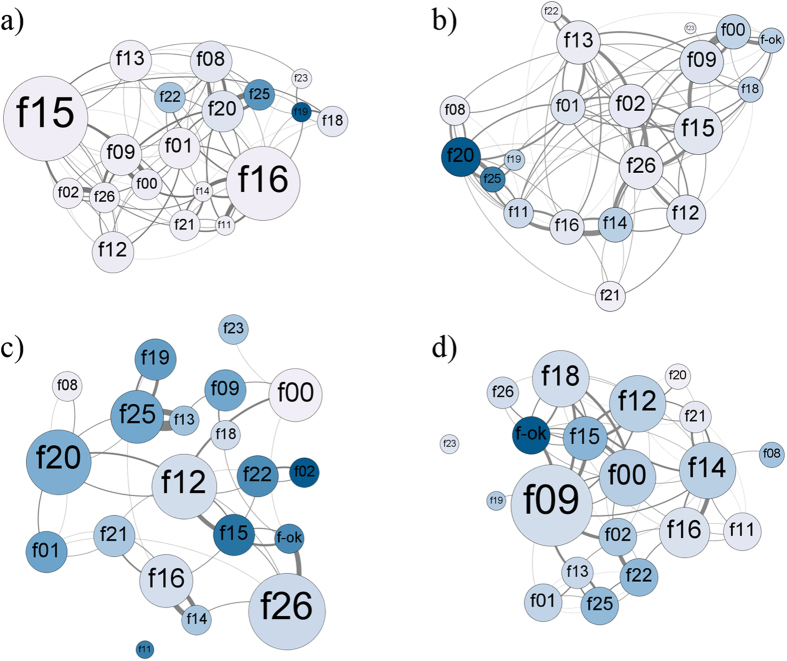
Interaction between centrality in grooming received network and seasonality in predicting lice load. Sociograms of weighted grooming received networks of adult female Japanese macaques of Koshima, divided according to season: (**a**) winter (N = 19, f-ok not followed), (**b**) spring (N = 20), (**c**) summer (N = 19, f11 no contact with others), and (**d**) fall (N = 19, f23 no contact with others). Variation in node colour represents variation in lice load per grooming unit, the darker the node, the higher the lice load. Variation in node size represents variation in degree, the bigger the node, the higher the degree. Variation in edge size represents the strength of interactions, the thicker the edge the more frequent grooming received between two nodes. Edge colour matches the target node, i.e. the node receiving grooming. A bidirectional relationship between two nodes is indicated by two edges clockwise.

**Figure 4:**
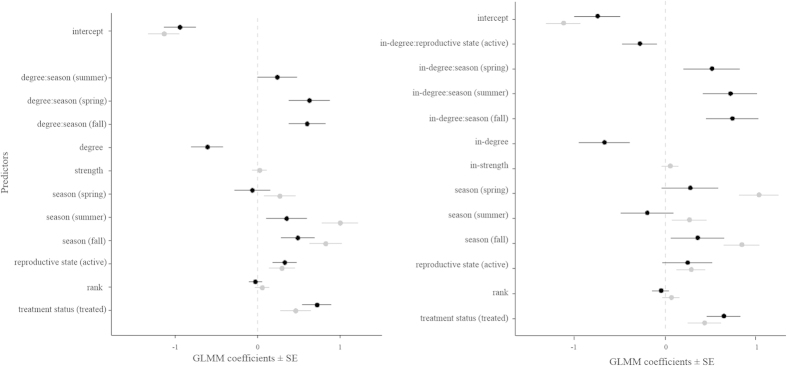
Coefficient plots of GLMMs. Testing prediction 1: left panel; Testing prediction 2: right panel. Circle: coefficient value, bold line: one standard error. Two variables separated by a column denotes an interaction. Levels of categorical predictors between parentheses indicate those not included in the intercept (the reference level is winter for season, not reproductively active for reproductive state, and non-treated for treatment). Black = degree (left) or in-degree (right). Grey = strength (left) or in-strength (right).

**Figure 5:**
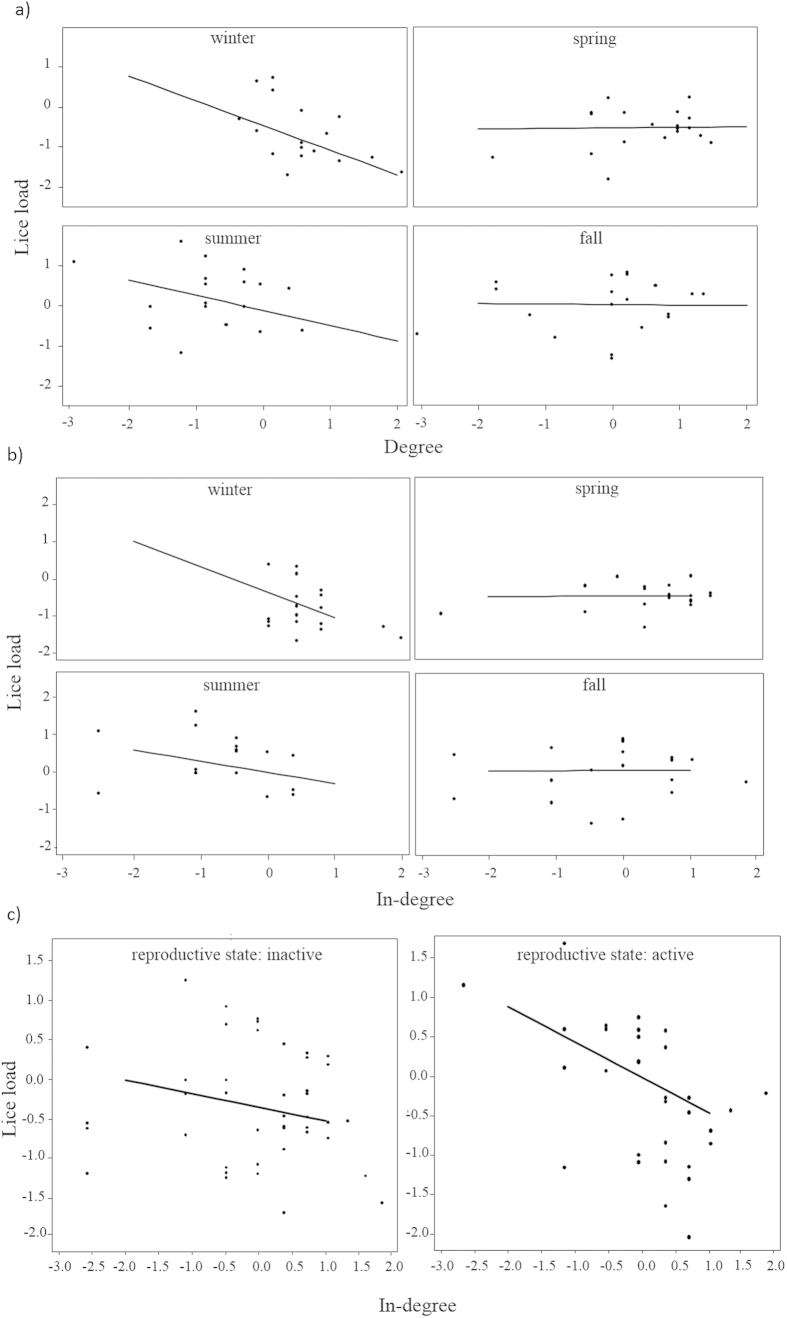
Effects of the interaction between number of connections in the network and seasonality or reproductive state on lice load. panel (**a**) shows degree (contact network) and season, panel (**b**) shows in-degree (grooming received network) and season, panel (**c**) shows in-degree (grooming received network) and reproductive state. The four seasons (winter, spring, summer, and fall) each have their own square, the two reproductive state (active, not active) as well. The line represents predictions from the model and dots the raw data transformed (i.e. log of the response (lice load) and square-root of the predictor (centrality)) and scaled.

Overall, only one of the centrality measures from contact networks was related to lice load and only through an interaction with season (Supplementary Tables S3 & S4, Figs 3, 4, 5): females with higher degree had a lower lice load than those with lower degree, but only in winter and summer, whereas there was no relationship between degree and lice load in spring and fall. Strength did not predict lice load (Supplementary Tables S3 & S4, Figs 3, 4, 5). Thus, in winter (mating season) and summer (birth season), females in contact with more female social partners had lower parasite burden compared to females in contact with fewer partners. In spring and fall, however, females showed similar parasite burden regardless of their centrality. The observed effect of degree centrality was significantly more pronounced than the same effect from a set of models that randomised the network data (p β_obs_ < β_rand_ = 0.043, Supplementary Table S4). Prediction 1 was thus not fulfilled.

### Testing prediction 2: decreased parasitism with increased centrality in grooming received network

Models with grooming received centrality measures showed a tendency towards lice load being related to in-degree through an interaction with season and reproductive state separately (Supplementary Table S3 & S4, Figs 3, 4, 5): females with higher degree in the grooming received network tended to have lower lice load than those with lower degree in winter and summer whereas there appeared to be no relationship between degree and lice load in spring and fall (Supplementary Table S3 & S4, Figs 3, 4, 5). This negative effect of centrality on lice load was marginally greater in reproductively active compared to inactive females (Supplementary Table S3 & S4, Figs 3, 4, 5). This means that in winter and summer (the mating and birth seasons respectively, i.e. when some females were reproductively active), females receiving grooming from more female social partners had lower parasite burden compared to females receiving grooming from fewer partners. In spring and fall, however, females showed similar parasite burden regardless of their centrality. However, the observed effect of in-degree centrality was not quite more pronounced than the same effect from a set of models that randomised the network data (p β_obs_ < β_rand_ p = 0.087, Supplementary Table S4). Prediction 2 was thus partially fulfilled.

## Discussion

In animal groups, increased centrality in social networks is often linked to increased parasite load and disease risk^3^. In female Japanese macaques of Koshima, centrality in terms of number of connections in contact and grooming received networks was negatively associated with lice load, as measured by louse egg-picking gestures performed during grooming. However, the relationship between degree and lice load was mediated by season in that females with fewer contact or grooming partners presented higher lice burden only during winter (mating season) and summer (birth season). This was concurrent with a tendency towards the negative effect of degree centrality in grooming received network on lice load to be greater in reproductively active compared to inactive females. These findings did not provide support to the prediction of increased parasitism with increased contact centrality, but were in accordance with other studies showing that grooming received can be a predictor of lower ectoparasite burden^15^,^19^,^20^,^47^.

It must be stated up front that our measure of lice load is indirect, observational and dependent upon observing individuals grooming. Capturing monkeys and marking/collecting lice provides a direct estimate of lice loads and allows testing whether lice actually carry pathogens, but this approach is inconvenient and often impossible to implement in wild populations. That said, Japanese macaques are very conspicuous when picking items from the hair or on the skin of their grooming partners, and 98.9% of what is picked and consumed has been demonstrated to be louse eggs^30^. Furthermore, body parts estimated to have many louse eggs are groomed longer with more frequent louse egg-picking gestures^17^,^30^. Thus, despite the indirect nature of this measure, we believe it to be a fair estimate of lice load, particularly when coupled with the demonstration that the amount of eggs is approximately double that of adult lice^17^. Such conspicuous hygiene-related behaviours thus provide useful information with which to investigate risks of infestation with ectoparasites and/or disease spread in wild animals, at least in those species in which such behaviours are readily observed.

The study of this louse egg-picking behaviour led researchers to discover that macaques groom for demonstrably longer durations if they find many louse eggs to pick and eat (so called “grooming-related feeding”), irrespective of the relationship between groomer and groomee^31^. Studies on birds have also shown that allopreening of self-inaccessible body parts occurred regardless of dominance relationships, which was not true for self-accessible body parts^15^. These studies suggest that some grooming bouts or parts thereof may be less dependent upon social preferences than once thought, and instead linked to other ecological functions (e.g. hygiene, feeding) of social grooming^15^,^31^. This is remarkable because social interactions between group members are often influenced by similarity within dyads; kin, individuals of adjacent dominance ranks, or age-mates tend to interact more frequently with each other than with others. Such assortativity may ultimately reflect on the centrality of individuals within their network, and arises independently of the distribution of lice in the group. Yet, our study, like that of earlier works^17^,^30^,^31^, hints at the possibility that grooming-related feeding may now prove influential in determining even partially the grooming network structure, at least in macaque societies, a link that should be investigated further.

One ecological factor that clearly affects numerous behavioural outcomes is seasonality. We considered seasonality of the environment as well as host and parasite biology, as it is generally highly relevant to infectious disease dynamics^32^. Indeed, the primary result of this study hinges on an interaction between seasonality, network centrality and lice load: the relationship between parasitism and sociality can only be interpreted in light of seasonal variation. Winter and summer represent the mating and birth seasons in Koshima, respectively. Both seasons show changes in female contacts and/or proximity behaviour^33^,^34^,^35^ in two opposite fashions: an increase as solitary/floater males temporarily join social groups to gain mating opportunities during the mating season^48^, and new individuals are born in the birth season; or conversely, a decrease as during the mating season, consortship pairs seek to be alone and during the birth season, females focus a great part of their attention on their newborns, somewhat decreasing their involvement in other social interactions^34^. These two seasons, winter (mating) and summer (birth), also have long-lasting effects on the physiology of females (e.g. immunosuppression, energy costs). It is therefore not surprising to observe that the greatest contrast observed in the relationship between lice load and centrality (from negative to almost absent) exist between the two most physiologically (reproductively) demanding seasons and the other less demanding seasons. Similarly, louse transfers and thus infection risk increased during the mating season in both chipmunks (*Tamias striatus)* and mouse lemurs *(Microcebus rufus)*, presumably because of the concomitant increase in both contact between individuals and host reproductive activity^28^,^49^. Because lice feed on blood, it has been hypothesised that their own reproduction is influenced by their hosts’ sexual hormones^21^,^23^, in that some stages of the host reproductive activity can trigger the parasite own reproductive activity. This is the case for another blood-feeding ectoparasite, the rabbit flea (*Spilopsyllus cuniculi)*, in which the reproductive cycle is triggered by pregnancy and parturition of the host doe. The fleas then migrate *en masse* to the nest to breed on the immunologically-naïve young^50^,^51^, illustrating the tight links between host and parasite biology and the effect of environmental seasonality on the synergy between host and parasite (see also next paragraph).

These synergies between host and parasite biology also explain the results of this study. Lice load was highest in summer during the periparturient period. Females thus seemed more vulnerable to louse infestation at this time. This was particularly the case for reproductively active females, especially those that were less central in the social networks. This pattern could be due to the combination of several factors, also linked to the effect of environmental seasonality on host and parasite biology. First, the surge of sex hormones in the host around birth could have triggered louse reproductive activity and proliferation^20^,^21^,^22^,^23^. Second, the immunosuppressive effects of these sex hormones during challenging times such as birth and lactation could have decreased the host’s defence against infestation, and made them more susceptible to it^52^. Third, the presence of immune-naïve hosts (newborns) could have constituted a newly available and easy breeding ground for lice, which could have then transferred to other individuals, especially vertically to the mother and then on to the mothers’ social partners. Fourth, moulting induces changes in the habitat of lice, and in Japanese macaques, begins at the onset of spring and ends during summer. Hairs are therefore shortest in summer and continue to grow through autumn and winter when they are longest. These changes, in addition to natural variations in the louse microhabitat (like skin temperature or solar radiation higher in summer^21^), likely impact louse prevalence and intensity (*sensu* the effect of shearing on louse density in domestic sheep^21^). We also cannot rule out the possibility that moulting impacts the efficiency with which monkeys can find and extract louse eggs: beginning at the hair base, louse eggs would be pushed upward during regrowth and the shorter hair may increase their visibility. Increases in lice load in summer through fall may thus be linked to the synergistic effects of changing louse habitat, the interaction between lice and host reproduction, and changes in behavioural tendencies of hosts. Nevertheless, examining factors related to seasonal variation in lice loads, including pelage characteristics, is insufficient in itself to explain the observed negative relationship between centrality and lice load during reproductive seasons.

At Koshima, mean centrality indices generally seemed to vary in the opposite direction of mean lice load across seasons; the season in which mean centrality was lowest corresponded to the season in which mean lice load was highest. However, network modularity did follow somewhat closer to the pattern of mean lice load in that the season in which mean lice load was highest was also the season in which modularity was greatest. Greater modularity in a network means that the network decomposes into more modules than in a network with lower modularity. Greater modularity is hypothesized to negatively impact transmission of infectious agents throughout social groups by breaking down the chain of transmission^53^. In our study, periods of increased lice load seemed concomitant with periods of higher social network modularity, reflecting either a decrease in females’ direct contacts and grooming exchanges or more focused exchanges within smaller cliques of individuals compared to periods with lower louse infestation. It seems likely, then, that decreases in grooming network centrality allow lice to multiply at both the individual and group levels, especially during periods of host immunological and physiological vulnerability (mating and birth seasons). Conversely, avoidance of social contacts is a well-known mechanism to limit the spread of socially-transmitted pathogens^53^,^54^. This requires that individuals can determine each other’s infection status, which seems likely in many animals^55^,^56^ but absent in others^57^. Of course, a third possibility is that changes in network structure and changes in lice load are concomitant but independent. While lice load may be environmentally determined, female hosts naturally increase social contacts to access mating opportunities and decrease them to care for their infants and minimise risks (of injury, disease), which may be stronger drivers of network changes compared to parasitism.

But if the network reflects pathways for parasite transmission, centrality indices such as degree and strength measuring risk of exposure are expected to be positively related to parasite infection patterns^3^. In our study, the negative relationship between degree and lice load during the winter and summer breeding seasons relative to spring and fall could suggest that individuals other than females have an impact on the lice burden or the likelihood of louse infestation during these periods: via vertical transmission between females and their immature offspring^21^ or horizontal transmission from solitary/floater males^58^. Barring this third-party influence, individual variation in grooming/contact given and received could also influence lice load, inasmuch as the presence of a “super-groomer” for example could play the role of a super-spreader–a highly contagious individual or simply one with many social connections^59^. Alternatively, such a super-groomer could play the role of “super-delouser”, as there seemed to be variation in louse egg-picking efficiency across individuals^44^, or if there is variation in grooming site preferences across individuals^17^. Ultimately, amongst the centrality measures we tested, the only predictor of lice load was the number of direct connections females had with others in the social network. This implies that infection risk was more related to who is connected to whom in the network rather than how individuals are connected. Thus, as was also shown in meerkats (*Suricata suricatta*)^60^, frequent social contact does not necessarily increase the risk of infection. From a parasite/pathogen perspective, one contact may be all it takes to change environment and continue reproducing on a new host. From a host perspective, having multiple social partners can either increase exposure to parasites through increased likelihood of interacting with a super-spreader, or increase parasite removal through increased likelihood of interacting with a super-delouser. These contradicting possibilities might also explain why observed relationships between centrality and lice load are not straightforward: grooming simultaneously offers lice a transmission opportunity and hosts a parasite removal opportunity. Our results are weighted toward the latter because less connected females generally exhibited higher louse burden, supporting the hypothesis that grooming is an effective antiparasite strategy to be exploited in a social context and providing further evidence for the benefits of being social despite the costs related to disease transmission and infection risk.

In this evolutionary arms race, parasites effectively use their hosts’ behaviour to increase their own fitness^1^, but hosts have evolved diverse social and ecological strategies of parasite avoidance and removal^61^. Due to the combined difficulties of accurately depicting animal contact networks from observation and of directly monitoring elusive parasite populations, experimental studies and/or alternative measures/estimations of both networks and parasites would help disentangling the synergistic effects of the environment and the interaction between host and parasite biology on transmission and infection risks from socially-transmitted pathogens^62^,^63^. For example, experimental reduction of parasite loads influences the frequency or patterns of host social interactions^64^,^65^. Conversely, manipulating host contact rates can also induce changes in infection risk^66^.

## Conclusion

In conclusion, our study shows that variation in contact and grooming network centrality in terms of number of connections explains variation in lice load in female Japanese macaques in that less central females have higher lice burden, providing further evidence of grooming as an efficacious anti-parasite strategy. However, our study also demonstrates that this relationship is dependent upon ecological and biological conditions, such as less central females have higher lice burden only during winter (cold–long, dense hair–mating season) and summer (hot–short, sparse hair–birth season). Our study is the first to estimate lice load from direct observation of wild animals and to link this estimate to the centrality status of individuals in their contact networks. This study also highlights the importance of seasonal variation both in parasite intensity and in host behaviour in explaining variance in infection risk and exposure from socially-transmitted external parasites.

## Methods

### Study site and subjects

We studied Japanese macaques on Koshima islet, Miyazaki prefecture, Japan (31°27′N, 131°22′E). The region has a warm-temperate climate with monthly mean temperatures ranging between 9.3 °C in January and 27.2 °C in August, and monthly mean precipitation between 35 mm in January (humidity 60%) and 730 mm in June (humidity 82%; data for 2014 from the Japan Meteorological Agency). Koshima is approximately 0.3 km^2^ and covered by evergreen broadleaved forest^67^. Provisioning and behavioural observations of Koshima macaques started in 1952, and demographic, ecological, behavioural, and life-history data have been collected since then^68^. The main group of Koshima macaques is currently provisioned with ca. 3 kg of wheat ca. twice weekly. Koshima is now inhabited by approximately 100 individuals divided into two social groups, Maki (ca. 15 individuals) and Main Arctic monkeys (ca. 60 individuals), along with an unknown number of solitary males. Monkeys are individually recognisable by facial tattoos and natural physical characteristics (scars, body shape or hair colour).

We observed the 19–20 adult females (>7 years old) of the Main group. We focused on females because, in macaque societies, they form the core of the group and dominate dynamics of social networks, males are often few and not very social, and juveniles are difficult to recognise, often have their own subgroup, and usually engage in different age-typical activities than the adults^69^,^70^,^71^. Ten females were in oestrous during the mating season (winter); they were thus considered reproductively active during this period. Seven females gave birth between the end of June and the beginning of July (summer), and they were considered reproductively active in spring (as pregnant), summer and fall (as lactating). All other females were regarded as non-reproductive for this year. Additionally, as part of an on-going research project since 2012, half of the adult female cohort is orally administered an anthelminthic treatment several times a year (MacIntosh, unpublished data). During the observation period, treatment was administered to 11 females at the end of March, the middle of July and the middle of November (see Supplementary Table S1 and the Statistics part for more details).

Research adheres to the ASAB/ABS guidelines for the use of animals in research and was approved by the ethic committee of Kyoto University Primate Research Institute.

### Data collection

Data were collected from January to November 2014 (total 142 days, 350 h of observation, 17h30 ± 1h15 per female, see Supplementary Table S1). Focal observations were balanced across females and time of day (morning/afternoon). To avoid the influence of artificial conditions on our data set, data other than dominance interactions were not collected during and up to an hour following provisioning. Focal females were followed for 15 min during which their main activities were recorded every minute, while their social, aggressive and other affiliative interactions as well as the identity of each social partner were recorded continuously. Amongst recorded activities, we distinguished between grooming given, grooming received, and simple body contact. We also collected data on dominance interactions (i.e. winner and loser of agonistic interactions) during focal observations and *ad libitum*.

During social and self-grooming bouts, we counted the number of times per minute-scan the groomer conspicuously picked out something in the groomee’s hair or her own and subsequently ate it. The gesture is conspicuous in the sense that the groomer will focus on a narrow patch of hair, pinch the base of the hair with the thumb and index fingers or her teeth, pull the selected object (a louse egg in 98.9% of the cases) along the length of the hair, and eat it^30^ (see Supplementary Video S1). If the focal female was the groomer and she picked louse eggs from the groomee, the louse egg counts were associated to the female groomee. If the focal female was the groomee, the louse egg counts were associated to her directly.

### Data analysis

We divided our dataset in four parts to account for seasonal variations biologically relevant to the studied host-parasite system. The winter dataset included observations between January and March and corresponded to the macaques’ mating season^72^, as well as to the period in which macaque hair is at its longest and densest^36^. The spring dataset included observations between April and June and corresponded to a non-breeding season and the period in which macaques started moulting. The summer dataset included observations between July and September and corresponded to the macaques’ birth season as well as the end of moulting, when the hair is at its shortest. Finally, the fall dataset included observations between October and November, and corresponded to a non-breeding season and a period in which hair resumes growth to its full length and density. For each season, we computed each female’s total number of scans of grooming received (including self-grooming), louse egg-picking gestures, centrality measures, dominance rank, reproductive state, and treatment status.

To compute lice load, we used minute-scans of grooming received, the focal female being either the groomer or the groomee, and minute-scans of self-grooming per female as well as minute-counts of louse egg-picking gestures across all grooming received bouts per female. Lice load was then calculated per female as follows:


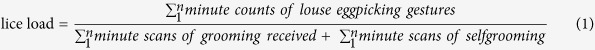


where n is the total number of grooming or self-grooming bouts.

We used SNA to investigate the risk of exposure to louse infestation of females within their social network of female group members. First, we calculated the modularity Q of the contact and grooming received networks (i.e., the number of clusters in the group) based on eigenvector centrality, which provides a descriptive statistic of detection and characterization of community structure in networks^73^, the higher it is the more clustered the network is. Here we use it to describe the global connectedness of the female group and track its changes across seasons because inter-individual distances seem to vary across seasons in Japanese macaques: females showed lower cohesiveness in summer^74^. We then computed individual indices of network centrality based on two behavioural datasets different from those used to estimate lice load; these data sets only included adult female-female interactions. The first dataset included dyadic total numbers of scans of general body contact, including grooming, between females A and B, which was used to build *undirected weighted networks of body contacts* (Fig. 1). The second dataset included dyadic total numbers of scans of grooming received from female A to female B and from female B to female A, which was used to build *directed weighted networks of grooming received* (Fig. 1). Although the two networks are not entirely independent, their actual differences in terms of number and strength of connections, and thus in terms of risk of exposure, are meaningful to test our predictions. From these networks, we then computed centrality indices reflecting only direct exposure to lice through the network as lice are exclusively transmitted through direct contact between hosts. Node *degree* reflected the number of direct connections an individual has in the network, and node *strength*, the sum of the weights of an individual’s direct connections (for a review, see)^3^.

To assign dominance rank, we calculated normalised David’s scores (normDS–package EloRating^75^), an individual score of relative power based on the successes (winning vs. losing) of an individual in agonistic interactions while accounting for the other group members’ successes^76^. We based our calculations on matrices of decided aggressive interactions, in which we could define a winner and a loser, and of displacements or supplantations recorded *ad libitum*. As there was no change in female hierarchical order across seasons, but individual normDS varied in magnitude, we finally assigned females an ordinal rank with 1 being the highest and 20 the lowest.

### Statistics

Centrality indices were calculated with the appropriate functions provided in the package igraph^73^,^77^. Network measures are not independent because they derive from a network where all individuals are linked to some extent and this non-independence violates many assumptions from most statistical tests. When testing the effect of factors on network measures or the effect of network measures on other variables this non-independence needs to be taken into account. A robust and modern standard way to do that is to compare statistical models based on the original observed data to a distribution of null models based on randomised data^78^,^79^. In this study, we randomised networks using the function rewire.edges of the package igraph, which rewires the end points (or nodes) of edges (i.e. edge rearrangement^78^) according to a probability of establishing connections that we set to vary randomly between 0 and 1 at each randomisation run. We used edge rearrangement to get a null model that randomly rearranges the observed interactions among pairs of nodes because we were confident in the observed edges and we constrained rearrangements by keeping the degree distribution of the original network to have a biologically meaningful null model^78^. Although the probability of a connection can be based on the number of individuals as potential partners, allowing the probability of establishing a connection to vary reflects natural processes of social partner choice. This approach thus provides a more conservative randomisation procedure. After each randomisation, network measures were recalculated and re-integrated in the statistical models (exactly the same models with all control factors but with the network measure derived from the randomisation). After 2000 randomisations, the statistical parameters of interest (e.g. model estimates or p values, see Supplementary Tables S2 & S4) were compared between models based on observed data and “null” models based on randomised data. If a substantial proportion (95%) of statistical parameters derived from models based on observed data were lower/higher than parameters derived from models based on randomised data, then we could conclude that the observed effects of or on sociality were different from those expected to arise by chance^78^,^79^. The randomisation procedure is exactly the same for all analyses.

To analyse relationships between the main variable of interest and the main predictor(s) while accounting for potential confounding factors, we built General Linear Mixed Models (GLMM) with Gaussian distributions and identity link functions using the function lmer from the lme4 package^80^. All models contained three main control factors: *dominance rank*, *reproductive state* and *season* (Table 1). In models with lice load as the response variable, an additional confounding factor, *treatment status* (binary: was/was not treated, Table 1), was included as the experimental anthelminthic treatment (Ivermectin) administered every 4 months to half the adult female cohort has been shown previously to affect louse numbers^81^. However, we observed only a small and transient decrease in estimated louse infestation approximately one week after treatment (unpublished data), and did not expect this result to have a significant effect on overall or seasonal lice load. Regardless, treatment was included in our models to control for these potentially confounding effects.

#### Assessment of the variation in centrality and lice load according to seasonal and individual factors

To assess the variability present in host sociality and lice load that may affect the relationship between the two, we built GLMMs with each centrality measure as the response variable, season, reproductive state and rank as predictors, and individual identity as random effects. Variation in lice load was assessed from the null model that was built to test predictions 1 and 2 and that included season, reproductive state, treatment status and individual identity as well (see below).

#### Relationship between measures of centrality and lice load: testing predictions 1 and 2

To address our main question concerning the relationship between measures of centrality and lice load, we built GLMMs with *lice load* as the response variable, *centrality index* as the main predictor, *season*, *reproductive state*, *rank*, and *treatment status* as confounding factors and *individual identity* as a random effect.

We also included a three-way interaction between centrality, season, and reproductive state, and its associated two-way interactions (centrality by season, centrality by reproductive state, and reproductive state by season). The effect of centrality on lice load could indeed depend on seasonal variations in both lice load and centrality, and lice load could also be differentially influenced by some stages of host reproduction (e.g., cycling versus lactating), and these stages depend on season. Thus, it may be that the effect of the interaction between centrality and season on lice load varies according to female reproductive state, such as females more central in the network have more lice in winter and summer but only if they are reproductively active (Table 1). We used likelihood ratio tests (LRT) to compare models with and without interactions and removed them if they did not improve model fit (at p LRT > 0.100). If the p-value of the LRT was between 0.100 and 0.050, we considered the interaction to marginally improve model fit and kept it in the model (Supplementary Table S3). Supplementary Table S3 shows that amongst all interactions tested, only those including centrality and either season or reproductive state remained in the models. Note that if an interaction was significant, predictors can be interpreted only within this interaction. We transformed (log or square-root) whenever necessary and then standardized (z-transformed) all numeric predictors for more accurate model fitting and ease of interpretation/comparison of model estimates. We checked several model assumptions (normality and homogeneity of residuals, variance inflation factors^82^) and no obvious violations or influential cases were detected. Because all centrality measures were correlated to each other to some extent (r range = 0.17–0.92, p range = 0.15–0.01), and because our dataset is comparatively small and thus could not accommodate their inclusion at once, we ran one model for each centrality measure. We tested final fitted models against a null model, comprising only control factors not involved in an interaction and the random effect, with a LRT. Control factors included in null models were not considered further. Whenever this test showed that adding predictors induced a significant improvement in model fit, we proceeded in interpreting the significance of the predictors. All statistics were done in R version 3.1.2^83^. The full results are given in the Supplementary Information (Supplementary Tables S2–4).

## Additional Information

**How to cite this article**: Duboscq, J. *et al.* Network centrality and seasonality interact to predict lice load in a social primate. *Sci. Rep.* **6**, 22095; doi: 10.1038/srep22095 (2016).

## Supplementary Material

Supplementary Information

Supplementary Video S1

## Figures and Tables

**Table 1:** Summary of control factors included in GLMMs: type, rationale, interactions between them and whether or not interactions contributed significantly to model fit (ns = not significant).

control factor	type	control for (rationale)	interactions between control factors	contributions of interaction to model fit (LR tests**)
season	categorical: winter, spring, summer, fall	host: moulting, network changes, physiological changes^32^,^33^,^34^,^35^,^36^,^72^,^74^ parasite: habitat change, fitness, population viability^21^,^23^,^32^	season* reproductive state* centrality	ns
reproductive state	categorical: active, not active	host: network changes, physiological changes^32^,^33^,^34^,^35^ parasite: fitness, population viability^20^,^21^	season* reproductive state	ns
rank	continuous	host: physical condition, access to grooming partners^9^,^37^,^38^,^43^ parasite: fitness, population viability^21^,^23^,^43^	repro* centrality	trend in model with in-degree as predictor
treatment*	categorical: treated, not treated	parasite: fitness^81^	season* centrality	significant in model with degree; trend in model with in-degree as predictors

^*^Factor only included in models with lice load as response variable.

^**^See Supplementary Table S2.
