# Supplementary material for: Network centrality and seasonality interact to predict lice load in a social primate
Source: Sci Rep. 2016 Feb 26;6:22095. doi: 10.1038/srep22095 (PMC4768153; doi:10.1038/srep22095)
Supplement: Supplementary Information [file srep22095-s1.pdf]

Supplementary material to the article:

Network centrality and seasonality interact to predict lice load in a social primate.

Julie Duboscq [1,2*], Valeria Romano [1,2], Cédric Sueur [1,2,3], and Andrew J.J. MacIntosh [4,5]

[1] Centre National de la Recherche Scientifique, Département Ecologie, Physiologie et Ethologie, Strasbourg, France

[2] Université de Strasbourg, Institut Pluridisciplinaire Hubert Curien, Strasbourg, France

[3] Unit of Social Ecology, CP231, Université libre de Bruxelles, Campus Plaine, Brussels, Belgium

[4] Kyoto University Primate Research Institute, Inuyama, Japan

[5] Kyoto University Wildlife Research Center, Kyoto, Japan

* Corresponding author: julie.a.m.duboscq@gmail.com

Methods

Table S1: Summary of the study females' characteristics (see main text).

female ID	observation time (hour)	rank	treatment status* (treated or non-treated)	reproductive state during the mating / other seasons (active or inactive)
f00	16.8	2	non-treated	inactive / inactive
f01	17.1	14	treated	active / active
f02	17.4	9	non-treated	active / active
f08	18.2	11	non-treated	active / active
f09	17.4	1	non-treated	active / active
f11	16.2	13	non-treated	active / active
f12	17.0	8	treated	inactive / inactive
f13	17.9	16	non-treated	active / active
f14	18.0	18	treated	inactive / inactive
f15	17.8	4	treated	inactive / inactive
f16	18.2	10	treated	inactive / inactive
f18	17.9	6	non-treated	active / inactive
f19	16.9	20	treated	inactive / inactive
f20	16.7	12	treated	inactive / inactive
f21	17.8	5	non-treated	active / inactive
f22	15.3	19	treated	active / inactive
f23	17.5	15	non-treated	inactive / inactive
f25	16.3	17	treated	active / active
f26	17.4	3	treated	inactive / inactive
f-ok	13.3	7	treated	no-data / inactive

* seasonal administration of anthelminthic treatment (Ivermectin)

Results

Table S2: Results of GLMMs testing the influence of seasonal and individual factors on centrality measures (contact network: degree, strength; grooming received network: in-degree, in-strength). Estimates and standard errors (β ± SE) with 95% confidence intervals (CI) are presented. Bold font indicates predictors with a significant effect on the response as indicated by CIs excluding 0. Parentheses indicate levels of categorical predictors not present in the intercept. Σ(p-val. obs.cent. < p-val. rand.cent.)/nb. rand. gives the number of times the p value of the LRT between full and null models with observed centrality was lower than the p value of the LRT between full and null models with randomised centrality divided by the number of randomisations (see main text). This gives a new p value, in bold when < 0.05.

	**Degree** (N = 79)	**Strength** (N = 79)	**In-degree** (N = 79)	**In-strength** (N =79)	**Lice load** (N = 79)
LRT full vs. null (χ^2, d.f., p)	**36.38, 5, <0.001**	**38.86, 5, <0.001**	**32.83, 5, <0.001**	**37.93, 5, <0.001**	**29.20, 5, <0.001**
Variables	β ± SE [95% CI]	β ± SE [95% CI]	β ± SE [95% CI]	β ± SE [95% CI]	β ± SE [95% CI]
intercept	0.55 ± 0.23 [0.09 1.08]	0.43 ± 0.23 [-0.04 0.91]	0.47 ± 0.22 [-0.03 0.90]	0.29 ± 0.22 [-0.15 0.72]	-0.83 ± 0.16 [-1.15 -0.49]
season (spring)	-0.09 ± 0.22 [-0.56 0.33]	0.07 ± 0.21 [-0.34 0.49]	-0.13 ± 0.24 [-0.61 0.34]	0.07 ± 0.21 [-0.34 0.44]	0.25 ± 0.19 [-0.12 0.61]
season (summer)	**-1.34 ± 0.23 [-1.82 -0.93]**	**-1.26 ± 0.22 [-1.67 -0.83]**	**-1.24 ± 0.24 [-1.73 -0.77]**	**-1.10 ± 0.21 [-1.52 -0.68]**	**0.95 ± 0.19 [0.55 1.35]**
season (fall)	**-0.73 ± 0.22 [-1.15 -0.30]**	**-0.58 ± 0.21 [-0.98 -0.17]**	**-0.72 ± 0.24 [-1.18 -0.24]**	**-0.55 ± 0.20 [-1.00 -0.18]**	**0.79 ± 0.20 [0.38 1.19]**
reproductive state (active)	-0.02 ± 0.25 [-0.54 0.48]	0.02 ± 0.25 [-0.45 0.53]	0.12 ± 0.23 [-0.32 0.52]	0.25 ± 0.24 [-0.22 0.72]	*0.15 ± 0.08 [-0.02 0.31]*
rank	-0.11 ± 0.14 [-0.37 0.13]	-0.16 ± 0.14 [-0.43 0.08]	**-0.28 ± 0.12 [-0.54 -0.04]**	**-0.33 ± 0.13 [-0.61 -0.05]**	0.15 ± 0.16 [-0.14 0.47]
Σ(p-val.obs.cent.<p-val.rand.cent.)/nb. rand.	**0.022**	0.185	0.570	0.941	not applicable

Table S3: Summary of likelihood ratio tests (LRT) between models with and without interactions and between the final model and an informative null model containing the two constant control factors, treatment and rank, plus season and/or reproductive state (in table "repro") if not included in an interaction. Results in **bold** indicate when the full model was a significantly better fit than the reduced one; results in *italics* indicate that the full model was only marginally (at p < 0.100) a significantly better fit but the interaction(s) had a significant effect on the response and was thus kept in the final model.

	**Centrality Index**	**Degree**	**Strength**	**In-degree**	**In-strength**
**full model**	**reduced model**	LRT full vs red.	LRT full vs red.	LRT full vs red.	LRT full vs red.
centrality*season*repro	season*repro<br>+ centrality*season<br>+ centrality*repro	χ^2=0.105<br>d.f.=3<br>p=0.991	χ^2=0.39<br>d.f.=3<br>p=0.606	χ^2=0.07<br>d.f.=3<br>p=0.995	χ^2=0.38<br>d.f.=3<br>p=0.336
season*repro<br>+ centrality*season<br>+ centrality*repro	centrality*season<br>+ centrality*repro	χ^2=0.232<br>d.f.=3<br>p=0.972	χ^2=1.18<br>d.f.=3<br>p=0.756	χ^2=0.39<br>d.f.=3<br>p=0.942	χ^2=3.41<br>d.f.=3<br>p=0.331
centrality*season<br>+ centrality*repro	centrality*season	χ^2=7.658<br>d.f.=1<br>p=0.103	χ^2=1.36<br>d.f.=1<br>p=0.243	*χ^2=3.32*<br>*d.f.=1*<br>*p=0.069*	χ^2=0.00<br>d.f.=1<br>p=0.994
centrality*season<br>+ centrality*repro	centrality*repro			*χ^2=6.97*<br>*d.f.=3*<br>*p=0.073*	
centrality*season	centrality<br>+ season<br>+ repro	**χ^2=9.436**<br>**d.f.=3**<br>**p=0.024**	χ^2=4.17<br>d.f.=3<br>p=0.243		χ^2=3.87<br>d.f.=3<br>p=0.275
centrality*repro	centrality<br>+ season<br>+ repro				

**final model**	centrality*season	centrality+season<br>+repro	centrality*season +<br>centrality*repro	centrality+season<br>+repro
**LRT final vs null model**	**χ^2=40.98**<br>**d.f.=7**<br>**p<0.001**	χ^2=0.05<br>d.f.=1<br>p=0.823	**χ^2=41.02**<br>**d.f.=9**<br>**p<0.001**	χ^2=0.31<br>d.f.=1<br>p=0.577

Table S4: Results of GLMMs testing the link between centrality in a contact (degree, strength) or grooming received (in-degree, in-strength) network and lice load. Estimates and standard errors (β ± SE) with 95% confidence intervals (CI) are presented. **Bold** font indicates predictors with a significant effect on the response within an interaction only or as a single effect, as indicated by CIs excluding 0. Single predictors included in interactions and control factors included in null models are not considered. Variables separated by a colon indicate an interaction. Parentheses indicate levels of categorical predictors not present in the intercept. Σ(β obs.cent. < β rand.cent.)/nb. rand. gives the number of times the observed estimate for centrality was lower than the randomised estimate divided by the number of randomisations (see main text). This gives a right p value that has been converted (between parentheses) into a left one, in bold when < 0.05, when the estimates were negative (then smaller is actually "bigger").

	**Degree** (N = 79)	**Strength** (N = 79)	**In-degree** (N = 79)	**In-strength** (N =79)
Variables	β ± SE [95% CI]	β ± SE [95% CI]	β ± SE [95% CI]	β ± SE [95% CI]
intercept	-0.93 ± 0.20 [-1.32 -0.54]	-1.13 ± 0.19 [-1.51 -0.75]	-0.81 ± 0.22 [-1.21 -0.32]	-1.13 ± 0.19 [-1.50 -0.74]
**centrality:season (spring)**	**0.63 ± 0.25 [0.14 1.12]**		**0.71 ± 0.30 [0.15 1.31]**	
**centrality:season (summer)**	0.24 ± 0.24 [-0.24 0.72]		0.48 ± 0.31 [-0.19 0.87]	
**centrality:season (fall)**	**0.60 ± 0.22 [0.16 1.04]**		0.72 ± 0.28 [-0.07 0.89]	
centrality: reproductive state (active)			*-0.27 ± 0.14 [-0.55 0.00]*	
centrality	-0.61 ± 0.20 [-0.99 -0.22]	0.02 ± 0.09 [-0.16 0.20]	-0.65 ± 0.28 [-1.22 -0.11]	0.05 ± 0.09 [-0.13 0.23]
season (spring)	-0.06 ± 0.22 [-0.49 0.36]	0.26 ± 0.19 [-0.11 0.64]	-0.09 ± 0.23 [-0.54 0.33]	0.26 ± 0.19 [-0.12 0.64]
season (summer)	0.35 ± 0.25 [-0.14 0.84]	1.00 ± 0.22 [0.56 1.43]	0.34 ± 0.26 [-0.19 0.87]	1.03 ± 0.21 [0.60 1.46]
season (fall)	0.49 ± 0.21 [0.07 0.90]	0.82 ± 0.20 [0.43 1.21]	0.41 ± 0.23 [-0.06 0.89]	0.84 ± 0.20 [0.45 1.23]
reproductive state (active)	0.33 ± 0.14 [0.03 0.62]	0.29 ± 0.16 [-0.02 0.61]	0.33 ± 0.15 [0.05 0.67]	0.27 ± 0.16 [-0.09 0.58]
rank	-0.03 ± 0.08 [-0.19 0.12]	0.04 ± 0.08 [-0.13 0.22]	-0.04 ± 0.09 [-0.24 0.15]	0.06 ± 0.09 [-0.12 0.25]
treatment status (treated)	0.71 ± 0.18 [0.36 1.06]	0.45 ± 0.19 [0.08 0.82]	0.63 ± 0.18 [0.26 0.99]	0.42 ± 0.18 [0.06 0.79]
Σ(βobs.cent.<βrand.cent.)/nb. rand.	**0.957 (p value left 0.043)**	0.548	*0.917 (p value left 0.087)*	0.447

Legend Video S1: A groomer parts the hair of a groomee with both hands, picks and eats a louse egg [6]. This is a typical example of louse egg-picking while grooming.
